# Sociodemographic and health factors associated with mortality in community-dwelling elderly

**DOI:** 10.1590/S1518-8787.2017051006708

**Published:** 2017-04-24

**Authors:** Flávia Silva Arbex Borim, Priscila Maria Stolses Bergamo Francisco, Anita Liberalesso Neri

**Affiliations:** I Faculdade de Ciências Médicas. Universidade Estadual de Campinas. Campinas, SP, Brasil

**Keywords:** Aged, Frail Elderly, Mortality, Risk Factors, Socioeconomic Factors, Gender and Health

## Abstract

**OBJECTIVE:**

The objective of this study is to identify factors associated with mortality, with emphasis on gender and age differences.

**METHODS:**

This is a cross-sectional study, which uses data from the FIBRA-2008-2009 network in Campinas, State of São Paulo, Brazil, with information on non-institutionalized residents of the urban area and the Mortality Information System. The dependent variable has been death, in 2013. The associations have been tested by odds ratio (OR) and their 95% confidence intervals, and the analyses have been conducted using the program Stata 12.0.

**RESULTS:**

Average age has been 72.3 years, 69.3% have been women, and 8.9% have died. We have found greater OR for mortality in individuals aged ≥ 75 years, classified as pre-frail or frail, and in those who have reported heart disease.

**CONCLUSIONS:**

In this study, the analysis of specific subgroups has allowed us to better understand the relationship between the factors associated with death in the elderly. With the exception of age, strategies based on primary and secondary care, focused on priority groups, can have a positive impact on the reduction of mortality among the elderly.

## RESUMO

**OBJETIVO:**

Identificar fatores associados à mortalidade, com ênfase nas diferenças de gênero e idade.

**MÉTODOS:**

Trata-se de estudo transversal, utilizando dados provenientes da rede FIBRA-2008-2009 em Campinas, SP, com informações de pessoas não institucionalizadas residentes na área urbana e pelo Sistema de Informações de Mortalidade. A variável dependente foi a ocorrência de óbito, verificada em 2013. As associações foram testadas pelas razões de chances (OR) e respectivos intervalos de confiança de 95%, e as análises conduzidas no programa Stata 12.0.

**RESULTADOS:**

A média de idade foi 72,3 anos, 69,3% eram mulheres e 8,9% foram a óbito. Encontrou-se maior OR para mortalidade nos indivíduos com idade ≥ 75 anos, nos classificados como pré-frágil ou frágil e naqueles que referiram doença do coração.

**CONCLUSÕES:**

Neste estudo, a análise para os subgrupos específicos permitiu melhor compreender a relação entre fatores que se associam ao óbito no idoso. Com exceção da idade, estratégias baseadas no cuidado específico de atenção primária e secundária, direcionadas a grupos prioritários, podem ter um impacto positivo na redução da mortalidade entre os idosos.

## INTRODUCTION

The combination of demographic and epidemiological transitions is associated with increased rates of mortality of elderly individuals in the general population. In fact, death in the elderly (aged 60 years or more) correspond to more than 60% of the total of deaths in the Brazilian population^10^, with emphasis on older and male elderly individuals. According to estimates published in 2007, 72% of the deaths of all Brazilians adults and elderly persons were due to chronic non-communicable diseases (NCD)^35^. If on the one hand that datum has a positive sense, on the other hand it indicates that public policies should invest even more strongly and consistently in strategies to prevent and promote health, with emphasis on health behaviors, such as how to improve the quality of life of the population of adults and elderly persons^36^.

The main causes of death in the elderly population are cardiovascular diseases (mainly ischemic heart disease and cerebrovascular^30^, neoplastic, and respiratory diseases)^10,23,28^. This pattern follows the prevalence of other countries^16^. In addition to chronic diseases, national and international studies investigate other variables of mortality risk in older adults, including the self-assessment of health, common mental disorder, functional capacity, number of drugs consumed, frailty, and falls^2,12,15,25,26^.

In Brazil, research studies examine the main causes of death from the records of the *Sistema de Informação de Mortalidade* (SIM – Mortality Information System), which allows us to know the epidemiological profile of death throughout the country. This system has been improved regarding the coverage and the quality of the data. A decreased number of variables with information that is ignored or not filled and a reduction of 53% in the percentage of deaths from ill-defined causes have enabled a better understanding on health and the transition of mortality^22,24^. However, the heterogeneity in the aging process still influences the accuracy in how health systems identify and record the main cause of death among the elderly, which contributes to the high correlation observed between the age above 65 years and the number of death records from ill-defined causes^22^.

For this population, the high prevalence of chronic diseases and the presence of multiple morbidities^5,33^, from the cumulative effects of exposure to stressors, indicate the need to consider the causes of mortality in specific subgroups, to guide health planning, actions, and strategies. The datum that living conditions harmful to health are especially present in adults and seniors most affected by economic inequality reinforces this idea^7^.

As death is not a repetitive event and cannot be attributed to a single risk factor, we need to consider the various concurrent and competitive risks that influence the adaptation of the elderly, and, therefore, we have carried out a study with records of the elderly, to verify the factors associated with mortality, with emphasis on gender and age differences.

## METHODS

This is a cross-sectional study which used the records on the elderly (aged 65 years or older) who participated in the FIBRA study, conducted in Campinas, State of São Paulo, Brazil, in 2008/2009.

To carry out the FIBRA Campinas research, we used a probabilistic sample (n = 900) by conglomerates, representative of the entire municipality, using census tracts of the urban area as sample unit. The elderly individuals were recruited in households by trained personnel. Recruitment and data collection were coordinated by the group that was formed to carry out the research and done in two successive steps. In each one, the elderly were recruited in numbers that would satisfy the estimates of the sampling plan for one or more adjacent census tracts and were forwarded to data collection^32^.

At the beginning of the single session of data collection, the elderly were invited to meet the conditions of the research and, if they agreed to participate, sign an informed consent. Each step was finished when the elderly had undergone an initial battery for frailty, anthropometric, clinical, sociodemographic, and mental status measurements. A score higher than the cutoff point in the Mini-Mental State Examination (MMSE), adjusted for years of schooling^8^, minus a standard deviation (SD), was the criterion used for the admission of the elderly as participants of the second battery of measurements, based on self-report instruments that assessed the following variables: chronic diseases, signs and symptoms, sleep problems, falls and fractures, use of drugs, visual and hearing impairment, smoking, alcoholism, and subjective health assessment; access to medical and hospital services in the last year; perception about oral health and functional conditions for eating; functionality indicated by the performance of advanced, instrumental and basic activities of daily life (AADL, IADL, and ADL, respectively); expectation of care; depressive symptoms and satisfaction^32^.

In this study we verified the occurrence of death among the elderly in 2013 in the SIM. To analyze the risk factors for death (yes or no), we extracted the variables of interest from the questionnaire of the FIBRA study, namely:

- Sociodemographic variables: gender (male, female), age (discrete values gathered into 65 to 74 and 75 years or more), and family income in minimum wages (MW) in force at the time of the research (values were grouped into ≤ 3 MW and > 3 MW).
- Self-reported chronic diseases: the information was obtained from dichotomous items (yes or no) that investigated if a doctor had diagnosed heart disease, hypertension, or diabetes mellitus sometime in the 12 months prior to the interview.
- Functional capacity: was evaluated from self-reports of the elderly regarding the help necessary for the performance of IADL and ADL. We considered as dependent the elderly who reported needing help partially or totally to carry out one or more ADL and IADL, according to the scale of Instrumental Activities of Daily Living of Lawton^4,21^, which includes seven practical life activities carried out in a close environment, and the scale of Activity of Daily Living of Katz^20^, which includes six activities associated with survival.
- Cognitive status: was evaluated using the Mini-Mental State Examination (MEEM), which consists of thirty items that evaluate the functions of temporal and spatial orientation, memory, attention, calculation, language, *praxias*, and visuoconstructive execution. As described earlier, the elderly who scored below the cutoff point for their education level, minus one standard deviation, were considered as having cognitive deficit suggestive of dementia.
- Geriatric Depression Scale (GDS-15): is a questionnaire with fifteen dichotomous items that evaluates dysphoric moods and feelings of helplessness, worthlessness, disinterest, boredom, and unhappiness in the past seven days. The cutoff point for Brazilian elderly individuals was estimated at 5 (considering as depressed the elderly with scores greater than this value), for sensitivity of 90.9% and specificity of 64.5%^3^. Self-assessment of health: was obtained from the question: “n general, you would say your health is: very good, good, regular, bad, or very bad?” For data analysis, we created two levels: very good/good *versus* regular/bad/very bad.
- Indicators of frailty: we considered the five criteria proposed by Fried et al.^14^ described below. Elderly persons qualified as frail had three or more criteria, the pre-frail scored one or two criteria, and the non-frail had no score points in any of the five criteria:
-
  - Unintentional weight loss in the last year (yes or no). In case of positive answer, we investigated the reduction (in kilograms), considering as positive the elderly who reported a loss greater than 4.5 kg or 5% of body weight.
  - Fatigue, measured by two self-report items obtained from the Center for Epidemiologic Studies Depression Scale (CESD), with four possibilities of answer (always, most of the time, few times, never or rarely). We considered as affirmative answer those who answered “always” or “most of the time” for any of the two questions.
  - Manual grip strength, measured with a Jamar dynamometer (Lafayette Instruments, Lafayette, Indiana, United States) placed in the dominant hand of the elderly, in three attempts, with a minute break between them. We considered as elderly with reduced force those whose average of the three measurements was among the 20% lowest distribution values, adjusted for gender and body mass index (BMI – weight/height^2^), according to the ranges suggested by the World Health Organization (WHO) and described by Marucci and Barbosa^29^. The cutoff points for the sample are as follows: men: 0 < BMI ≤ 23, cutoff point (CP) ≤ 27.00 kgf; 23 < BMI < 28, CP ≤ 28.67 kgf; 28 ≤ BMI < 30, CP ≤ 29.50; BMI ≥ 30, CP ≤ 28.67; women: 0 < BMI ≤ 23, CP ≤ 16.33; 23 < BMI < 28, CP ≤ 16.67; 28 ≤ BMI < 30, CP ≤ 17.33; BMI ≥ 30, CP ≤ 16.67.
  - Gait speed, indicated by the average time in seconds spent by the elderly to go a distance of 4.6 meters, three times, in their usual speed, in a flat terrain, according to the recommendations of Guralnik et al.^18^ and Nakano et al.^31^ We considered as elderly with reduced speed those whose average of three measurements was among the 20% highest distribution values for time, in seconds, in relation to time that the elderly of the sample needed to accomplish the task. The averages were adjusted by the median height for men and women (men: 0 < height ≤ 168 cm, CP ≤ 5.49 seconds; height > 168 cm, CP ≤ 5.54 seconds; women: 0 < height ≤ 155 cm, CP ≤ 6.61 seconds; height > 155 cm, CP ≤ 5.92 seconds).
  - Physical activity: corresponds to the weekly frequency and daily duration of physical exercise, sports, and household chores, based on the answers to the items of the Minnesota Leisure Time Activity Questionnaire^14,27,39^. To calculate the weekly caloric expenditure on leisure activities and on household chores, we considered the number of items to which the elderly replied affirmatively, multiplied by the number of days in the week and the number of minutes per day that the activities were practiced. Then we calculated the quintiles of the distribution of this variable for men and women separately. We considered as inactive the elderly who scored among the 20% lowest distribution values for weekly caloric expenditure for their gender.
- Number of falls: we gathered data on it using a dichotomous question that asked whether the elderly had fallen in the last 12 months. For those who responded affirmatively, we asked how many times they had fallen. Answers were categorized into “never fell” and “one or more falls”.

The FIBRA protocols were successively checked by two supervisors. The checking of the data, in electronic database, was performed by two trained evaluators, with a requirement of 100% agreement.

The characterization of the sample was made by calculating the absolute and relative frequencies of the variables considered. Initially, we verified the association between the various variables and the outcome (death) by odds ratio (OR) and their 95% confidence intervals. Considering the effect of gender and age on the risk of death, we calculated the adjusted odds ratios and, in the adjustment, we also considered the variable of per capita household income – proxy for socioeconomic level.

We also estimated the cumulative proportion of deaths in the period and the odds ratio adjusted according to gender and age groups. Then, we carried out the hierarchical multiple logistic regression analysis. In the first step, we included the variables of level 1 (gender, age group, and per capita income), which remained in the model, regardless of statistical significance, as adjustment variables^7^(Figure). In the second step, we inserted the other variables and kept those that were adjusted by the variables of level 1 or those that presented p < 0.05.

**Figure:**
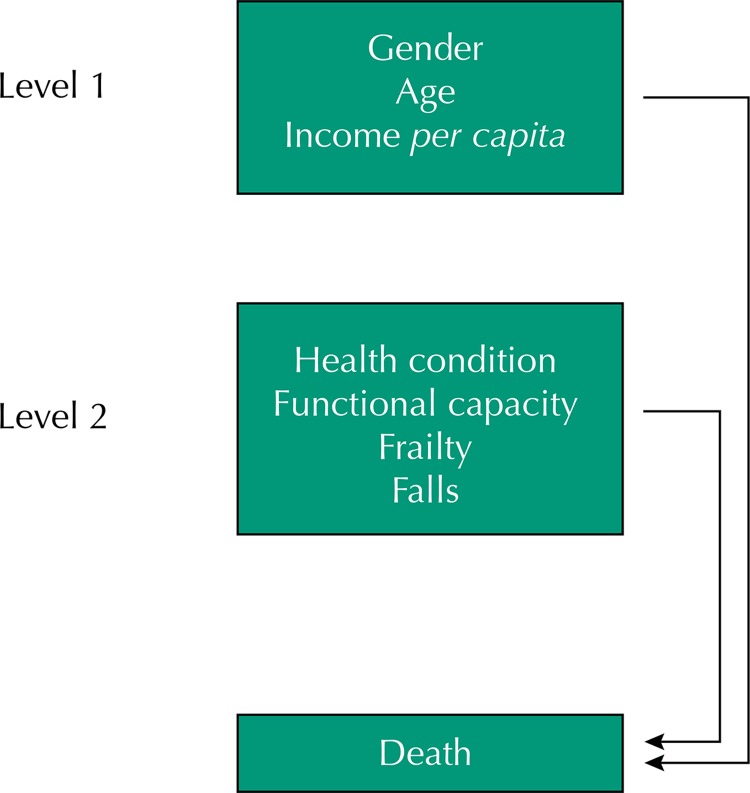
Theoretical model for the investigation of risk factors for mortality in the elderly, structured into hierarchical levels.

## RESULTS

The average age of the elderly was 72.3 years (SD = 5.4) and maximum age was 90 years; 69.3% were women and 8.9% died. Increased odds ratios for mortality were observed for the categories of 75 years or more, presence of heart disease, and classification as pre-frail or frail (Table 1).

**Table 1:** Percentage distribution of the sample, proportion of deaths, and odds ratio for mortality, according to sociodemographic variables, health condition, functional capacity, frailty, and falls. Study FIBRA Campinas, 2008-2009.

Variable	n (%)	Mortality		

		%	_Crude_OR (95%CI)	_Adjusted_OR^a^ (95%CI)
Gender^b^		*p = 0.100*		
Female	*624 (69.3)*	*7.8*	1	1
Male	*276 (30.7)*	*11.2*	1.48 (0.92–2.38)	1.54 (0.95–2.50)
Age group (years)^c^		p = 0.000		
65–74	*595 (66.1)*	*6.4*	1	1
≥ 75	*305 (33.9)*	*13.7*	2.34 (1.47–3.71)	2.37 (1.48–3.79)
Income (minimum wage)^d^		*p = 0.433*		
> 3	*386 (42.9)*	*8.0*	1	1
≤ 3	*514 (57.1)*	*9.5*	1.20 (0.75–1.93)	1.12 (0.69–1.81)
Hypertension		*p = 0.439*		
No	246 (35.6)	9.4	1	1
Yes	444 (64.4)	7.7	0.80 (0.46–1.40)	0.86 (0.49–1.51)
Diabetes		*p = 0.630*		
No	538 (78.0)	8.0	1	1
Yes	152 (22.0)	9.2	1.16 (0.62–2.19)	1.13 (0.59–2.16)
Heart disease		p = 0.027		
No	508 (73.7)	6.9	1	1
Yes	181 (26.3)	12.2	1.87 (1.06–3.28)	1.87 (1.05–3.31)
Functional capacity		*p = 0.170*		
Independent	464 (68.8)	7.3	1	1
Dependent	210 (31.2)	10.5	1.48 (0.84–2.60)	1.32 (0.73–2.38)
Cognitive status		*p = 0.235*		
No deficits	688 (76.6)	8.3	1	1
With deficits	210 (23.4)	11.0	1.36 (0.81–2.27)	1.06 (0.62–1.82)
GDS		*p = 0.695*		
≤ 5 depressive symptoms	547 (80.6)	8.0	1	1
≥ 6 depressive symptoms	132 (19.4)	9.1	1.14 (0.58–2.23)	1.13 (0.57–2.25)
Self-assessment of health		*p = 0.473*		
Excellent/Very good	405 (58.9)	7.7	1	1
Regular/Bad/Very bad	283 (41.1)	9.2	1.22 (0.70–2.10)	1.31 (0.75–2.29)
Frailty		p = 0.010		
Not frail	360 (40.1)	5.8	1	1
Pre-frail and frail	538 (59.9)	10.8	1.95 (1.16–3.27)	1.70 (1.00–2.91)
Number of falls		p = 0.573		
Zero	474 (73.6)	8.0	1	1
1 or more	170 (26.4)	9.4	1.19 (0.64–2.20)	1.12 (0.59–2.14)

GDS: Geriatric Depression Scale

^a^ OR adjusted by gender, age, and income.

^b^ OR adjusted by age and income.

^c^ OR adjusted by gender and income.

^d^ OR adjusted by age and income.

Values in italic: value of p ≤ 0.05.

Values in bold: OR greater than the reference category.

Table 2 shows the results of the proportion of deaths according to gender. We did not observe associations in females, between the variables considered and the deaths in this subgroup. For males, we observed greater odds ratio for mortality in the elderly who reported falling in the last year.

**Table 2:** Proportion of death by gender, according to variables of health condition, functional capacity, frailty, and falls. Study FIBRA Campinas, 2008-2009.

Variable	Mortality			

	Female		Male	
	
	%	_Adjusted_ OR* (95%CI)	%	_Adjusted_ OR* (95%CI)
Hypertension	*p = 0.416*		*p = 0.975*	
No	8.4	1	11.0	1
Yes	6.3	0.84 (0.40–1.76)	10.8	0.93 (0.39–2.23)
Diabetes	*p = 0.983*		*p = 0.538*	
No	7.0	1	10.2	1
Yes	7.1	1.14 (0.47–2.76)	13.2	1.16 (0.44–3.04)
Heart disease	*p = 0.124*		*p = 0.135*	
No	6.0	1	8.9	1
Yes	10.1	1.82 (0.85–3.90)	15.8	1.95 (0.81–4.72)
Functional capacity	*p = 0.111*		*p = 0.726*	
Independent	5.8	1	10.4	1
Dependent	9.9	1.51 (0.71–3.22)	12.0	1.02 (0.38–2.68)
Cognitive status	*p = 0.255*		*p = 0.598*	
No deficits	7.2	1	10.7	1
With deficits	10.1	1.10 (0.56–2.17)	13.1	0.91 (0.36–2.24)
GDS	*p = 0.984*		*p = 0.416*	
≤ 5 depressive symptoms	7.1	1	10.0	1
≥ 6 depressive symptoms	7.1	1.02 (0.42–2.52)	14.7	1.36 (0.46–4.04)
Self-assessment of health	*p = 0.443*		*p = 0.776*	
Excellent/Very good	6.2	1	10.5	1
Regular/Bad/Very bad	8.1	1.45 (0.69–3.05)	11.7	1.17 (0.49–2.82)
Frailty	*p = 0.108*		p = 0.022	
Not frail	5.5	1	6.4	1
Pre-frail and frail	9.0	1.31 (0.66–2.61)	15.2	2.29 (0.97–5.40)
Number of falls	p = 0.649		p = 0.020	
Zero	7.7	1	8.6	1
1 or more	6.4	0.71 (0.31–1.63)	22.6	3.25 (1.14–9.22)

GDS: Geriatric Depression Scale

* OR adjusted by age and income.

Values in italic: value of p ≤ 0.05.

Values in bold: OR greater than the reference category.

In relation to the proportion of deaths by age group, among the elderly aged 75 years or more, there were no significant differences in relation to deaths and the independent variables studied. In the elderly aged 65 to 74 years, higher death rates were observed in those who reported presence of heart disease and cognitive deficits (Table 3).

**Table 3:** Proportion of death by age group, according to variables of health condition, functional capacity, frailty, and falls. Study FIBRA Campinas, 2008-2009.

Variable	Mortality			

	65-74 years		75 years or more	
	
	%	_Adjusted_ OR* (95%CI)	%	_Adjusted_ OR* (95%CI)
Hypertension	*p = 0.713*		*p = 0.254*	
No	*4.9*	1	18.0	1
Yes	*5.7*	1.28 (0.54–3.05)	12.4	0.63 (0.29–1.37)
Diabetes	*p = 0.655*		*p = 0.190*	
No	*5.7*	1	13.0	1
Yes	*4.6*	0.79 (0.29–2.17)	20.9	1.64 (0.68–3.94)
Heart disease	p = 0.005		*p = 0.671*	
No	3.7	1	14.0	1
Yes	10.3	2.94 (1.32–6.56)	16.3	1.16 (0.50–2.72)
Functional capacity	*p = 0.677*		*p = 0.434*	
Independent	5.2	1	13.1	1
Dependent	6.2	1.22 (0.50–2.96)	17.1	1.34 (0.60–2.97)
Cognitive status	p = 0.045		*p = 0.418*	
No deficits	5.4	1	14.9	1
With deficits	10.5	2.07 (1.00–4.30)	11.4	0.71 (0.34–1.50)
GDS	*p = 0.324*		*p = 0.173*	
≤ 5 depressive symptoms	6.0	1	12.7	1
≥ 6 depressive symptoms	3.4	0.56 (0.16–1.94)	20.9	1.76 (0.72–4.33)
Self-assessment of health	*p = 0.240*		*p = 0.874*	
Excellent/Very good	4.4	1	14.4	1
Regular/Bad/Very bad	6.8	1.62 (0.73–3.60)	15.2	1.02 (0.46–2.28)
Frailty	*p = 0.231*		*p = 0.092*	
Not frail	5.1	1	8.2	1
Pre-frail and frail	7.5	1.52 (0.77–3.01)	15.6	2.26 (0.95–5.38)
Number of falls	*p = 0.625*		*p = 0.452*	
Zero	5.8	1	13.8	1
1 or more	4.6	0.88 (0.31–2.45)	18.0	1.39 (0.59–3.26)

GDS: Geriatric Depression Scale

* OR adjusted by gender and income.

Values in italic: value of p ≤ 0.05.

Values in bold: OR greater than the reference category.

From the multiple logistic regression model, we found, in the first step, greater odds ratio for mortality in men and the elderly aged 75 years or more; in the second step, those classified as pre-frail or frail presented greater odds ratio for death in relation to non-frail (OR = 1.89; 95%CI 1.02–3.50), and for heart disease, the greatest odds ratio was on the threshold of statistical significance (p = 0.055) (Table 4).

**Table 4:** Hierarchical regression model for mortality, according to sociodemographic variables, health condition, functional capacity, frailty, and falls. Study FIBRA Campinas, 2008-2009.

Variable	Mortality	

	First step^a^	Second step^b^
	
	_Adjusted_ OR (95%CI)	_Adjusted_ OR (95%CI)
Gender		
Female	1	
Male	1.55 (0.96–2.52)	
Age group (years)		
65–74	1	
≥ 75	1.57 (1.26–1.96)	
Income (minimum wage)		
> 3	1	
≤ 3	1.12 (0.70–1.81)	
Frailty		
Not frail		1
Pre-frail and frail		1.89 (1.02–3.50)
Heart disease		
No		1
Yes		1.76 (0.98–3.14)

_Adjusted_ OR: adjusted odds ratio by multiple logistic regression (688 individuals were included in the final model)

^a^ Adjusted by gender, age, and income.

^b^ Adjusted by sociodemographic variables, health condition, functional capacity, frailty, and falls.

Values in bold: OR greater than the reference category.

## DISCUSSION

This study sought to identify the factors associated with mortality, with emphasis on gender and age differences, five years after the completion of the FIBRA Research, performed in 2008/2009 in the city of Campinas.

In relation to gender, associations were not observed between the variables studied and death in women. Women are more careful and go more often after health services than men, which would explain in part the lower frequency of deaths among elderly women than among elderly men^9,34,37^. Nevertheless, we need a more adequate healthcare for elderly women, in order to prevent and delay health problems and promote their quality of life. Among men, only the occurrence of falls increased the chance of death. A study in Rio Grande do Sul, Brazil, which has analyzed death by falls in the elderly, has observed that men showed greater mortality coefficients for falls in relation to women for the age range from 60 to 79 years^34^. For men, it is crucial the increase of the access to health services, especially in the identification of markers that increase the possibility of falls, in addition to the need to intensify the control of risk factors and actions for the prevention of the severity of injuries, with early diagnosis and treatment.

Regarding deaths, according to age groups, the chance is higher among individuals who reported heart disease and among those who presented cognitive deficit. Cognitive deficit and heart disease are variables that are associated with aging^13^. The proper control and treatment of health problems, with emphasis on greater access to primary health care and change in lifestyle, must be a priority of the health system, with the adoption of measures for the evaluation, diagnosis, and intervention of these problems, in order to identify treatable causes and extend the independence, autonomy, and life expectancy of the elderly.

As for income, which has many associations with health variables, the literature shows that in all age groups and, mainly, in the most advanced ages, persons with lower socioeconomic status have worse health conditions^34,37^. Health is associated with individual characteristics and the characteristics of the community where the person lives. Thus, even though, in this study, they do not present a statistically significant association with death, we must consider that socioeconomic indicators are important variables, because they reflect conditions that influence health behaviors, self-care, and health condition of the individual^6^.

There was no significant difference in the proportion of deaths observed between men and women. A study that has described associations controlled by gender between mortality and NCD has observed that mortality by NCD is considerably higher in men than in women^38^, despite the decline in the rates of both genders, noted in the last two decades. Lifestyle and the demand and use of health services for prevention and assistance are aspects that contribute to the increased mortality in men. In this sense, we highlight the health inequality according to gender and the need for interventions able to ensure the differentiated confrontation of risk factors for health diseases and problems among men and women^1^.

Mortality was 76% higher among those who reported heart disease than among those who did not report it. It is known that diseases of the circulatory system are still the leading cause of death among Brazilian elderly individuals, despite the reduction of the mortality coefficient for cardiovascular diseases in both genders and in all the ages^11^ in the last 15 years. Of the causes of avoidable death, chronic diseases account for 82.6% of them, and, among them, heart diseases had the highest percentage (56.6%)^19^.

Frailty syndrome is usually described as a condition of increased vulnerability expressed in reduced compensatory responses and the possibility of maintaining homeostasis in relation to stressors, which results in increased adverse health outcomes, such as falls, disability, hospitalization, and death^14^. There is no consensus regarding the operational definition of the construct, but generally it does not concern only the biological or physiological determinants, but a multidimensional condition that involves the physical, psychological, and social domains^9^.

This study showed greater odds ratio for mortality in pre-frail and frail elderly (OR = 1.89; 95%CI 1.02–3.50), corroborating previous studies^14,15,17,28^. The results of the analysis between frailty and the variable gender indicated greater odds ratio for mortality in men. Although women present higher prevalence of isolated criteria of frailty when compared to men (for example, 62.1% of women were pre-frail or frail, while 54.9% of the men were in this category), increased mortality was observed among pre-frail and frail men. Thus, we can say that clinical attention should be directed at the early detection of frailty among men. Control of risk factors, as well as proper intervention and rehabilitation, can slow adverse health outcomes, especially mortality. Studies should be conducted to better understand effective interventions in the prevention and improvement of frailty and others to understand the benefits and risks of potential clinical interventions.

We have been careful to prevent systematic distortion of the data, by encouraging the participation of the elderly, standardizing the procedures, instruments, and equipment, and by extensively training the teams of recruitment and data collection, in addition to using procedures to ensure greater reliability of the data entered in electronic databases. Nevertheless, the limitations resulting from the design contraindicate broad generalizations. Among the limitations, we can mention the exclusion of bedridden and institutionalized elderly persons, which may have led to the underestimation of the mortality rate. We highlight that the SIM data are subject to underreporting for various reasons associated with the organization of health services. In addition, the SIM does not record the death of elderly individuals from the municipality which occurred outside of its coverage area. It is possible that the total number of deaths in the period have been insufficient to verify the associations intended for some of the variables investigated, such as gender.

It is known that gender and age differentials and socioeconomic and cultural characteristics, as well as the characteristics related to subjective indicators and the indicators of access to health services, change the magnitude of the risk for many diseases and mortality. In this study, the analysis of specific subgroups allowed us to better understand the relationship between the factors associated with death in the elderly. With the exception of age, strategies based on primary and secondary care, focused on priority groups, can have a positive impact on the reduction of mortality among the elderly.

## References

[1] Abreu DMX, César CC, França EB (2009). Diferenciais entre homens e mulheres na mortalidade evitável no Brasil (1983-2005). Cad Saude Publica.

[2] Alamgir H, Muazzam S, Narrullah M (2012). Unintentional falls mortality among elderly in the United States: time for action. Injury.

[3] Almeida OP, Almeida SA (1999). Short versions of the Geriatric Depression Scale: a study of their validity for the diagnosis of major depressive episode according to ICD-10 and DSM-IV. Int J Geriatr Psychiatry.

[4] Andreotti R, Okuma SS (1999). Tradução, adaptação transcultural e análise das propriedades psicométricas do Questionário Minnesota de Atividades Físicas e de Lazer.

[5] Batista SR (2014). A complexidade da multimorbidade. J Manag Prim Health Care.

[6] Bell ML, Zanobetti A, Dominici F (2013). Evidence on vulnerability and susceptibility to health risks associated with short-term exposure to particulate matter: a systematic review and meta-analysis. Am J Epidemiol.

[7] Belon AP, Barros MBA, Marin-León L (2012). Mortality among adults: gender and socioeconomic differences in a Brazilian city. BMC Public Health.

[8] Brucki SMD, Nitrini R, Caramelli P, Bertolucci PHF, Okamoto IH (2003). Sugestões para o uso do mini-exame do estado mental no Brasil. Arq Neuropsiquiatr.

[9] Buckinx F, Rolland Y, Reginster JY, Ricour C, Petermans J, Bruyère O (2015). Burden of frailty in elderly population: perpectives for a public health challenge. Arch Public Health.

[10] Cabrera MAS, Andrade SM, Wajngarten M (2007). Causas de mortalidade em idosos: estudo de seguimento de nove anos. Geriatr Gerontol.

[11] Carvalho MHR, Carvalho SMR, Laurenti R, Payão SLM (2014). Tendência de mortalidade de idosos por doenças no município de Marília- SP, Brasil: 1998 a 200 e 2005 a 2007. Epidemiol Serv Saude.

[12] Dapp U, Minder CE, Anders J, Golgert S, Renteln-Kruse W (2014). Long-term prediction of changes in health status, frailty, nursing care and mortality in community-dwelling senior citizens: results from the longitudinal urban cohort ageing study (LUCAS). BMC Geriatr.

[13] Ferreira PCS, Tavares DMS, Rodrigues RAP (2011). Características sociodemográficas, capacidade funcional e morbidades entre idosos com e sem declínio cognitivo. Acta Paul Enferm.

[14] Fried LP, Tangen CM, Walston J, Newman AB, Hirsch C, Gottdiener J (2001). Frailty in older adults: evidence for a phenotype. J Gerontol A Biol Sci Med Sci.

[15] Garre-Olmo J, Calvó-Perxas L, López-Pousa S, Gracia-Blanco M, Vilalta-Franch J (2013). Prevalence of frailty phenotypes and risk of mortality in a community-dwelling elderly cohort. Age Ageing.

[16] GBD 2013 Mortality and Causesof Death Collaborators (2015). Global, regional, and national age-sex specific all-cause and cause-specific mortality for 240 causes of death, 1990-2013: a systematic analysis for the Global Burden of Disease Study 2013. Lancet.

[17] Graham JE, Snih SA, Berges IM, Ray LA, Markides KS, Ottenbacher KJ (2009). Frailty and 10-year mortality in community-living Mexican American older adults. Gerontology.

[18] Guralnik JM, Simonsick EM, Ferrucci L, Glynn RJ, Berkman LF, Blazer DG (1994). A short physical performance battery assessing lower extremity function: association with self-reported disability and prediction of mortality and nursing home admission. J Gerontol Med Sci.

[19] Kanso S, Romero DE, Leite IC, Marques A (2013). A evitabilidade de óbitos entre idosos em São Paulo, Brasil: análise das principais causas de morte. Cad Saude Publica.

[20] Katz S, Ford AB, Moskowitz RW, Jackson BA, Jaffe MW (1963). Studies of illness in the aged. The index of ADL: a standardized measure of biological and psychosocial function. JAMA.

[21] Lawton MP, Brody EM (1969). Assessment of older people: self-maintaining and instrumental activities of daily living. Gerontologist.

[22] Lima EEC, Queiroz BL (2014). Evolution of the deaths registry system in Brazil: associations with changes in the mortality profile, under-registration of death counts, and ill-defined causes of death. Cad Saude Publica.

[23] Lima-Costa MF, Peixoto SV, Giatti L (2004). Tendências da mortalidade entre idosos brasileiros (1980-2000). Epidemiol Serv Saude.

[24] Lima-Costa MF, Matos DL, Laurenti R, Mello-Jorge MHP, Cesar CC (2010). Time trends and predictors of mortality from ill-defined causes in old age: 9 year folllow-up of the Bambuí cohort study (Brazil). Cad Saude Publica.

[25] Lima-Costa MF, Peixoto SV, Matos DL, Firmo JOA, Uchôa E (2011). Predictors of 10-year mortality in a population of community-dwelling Brazilian Elderly: the Bambuí Cohort Study of Aging. Cad Saude Publica.

[26] Llibre JJ, López AM, Valhuerdi A, Guerra M, Llibre-Guerra JJ, Sánchez YY (2014). Frailty, dependency and mortality predictors in a cohort of Cuban older adults, 2003-2011. MEDICC Rev.

[27] Lustosa L, Pereira D, Dias R, Britto R, Pereira L (2010). Tradução, adaptação transcultural e análise das propriedades psicométricas do Questionário Minnesota de Atividades Físicas e de Lazer.

[28] Maia FOM, Duarte YAO, Lebrão ML (2006). Análise dos óbitos em idosos no Estudo SABE. Rev Esc Enferm USP.

[29] Marucci M, Barbosa A, Lebrão ML, Duarte YAO (2003). Estado nutricional e capacidade física. SABE – Saúde, Bem-estar e Envelhecimento: o Projeto SABE no município de São Paulo: uma abordagem inicial.

[30] Ministério da Saúde (BR), Secretaria de Vigilância em Saúde, Departamento de Análise de Situação de Saúde, Coordenação Geral de Doenças e Agravos Não Transmissíveis (2011). Plano de ações estratégicas para o enfrentamento das doenças crônicas não transmissíveis (DCNT) no Brasil 2011-2022.

[31] Nakano MM (2007). Adaptacão cultural do instrumento Short Physical Performance Battery - SPPB: adaptação cultural e estudo da confiabilidade.

[32] Neri AL, Yassuda MS, Araújo LF, Eulálio MC, Cabral BE, Siqueira MEC (2013). Metodologia e perfil sociodemográfico, cognitivo e de fragilidade de idosos comunitários de sete cidades brasileiras: Estudo FIBRA. Cad Saude Publica.

[33] Oliveira TC, Medeiros WR, Lima KC (2015). Diferenciais de mortalidade por causas nas faixas etárias limítrofes de idosos. Rev Bras Geriatr Gerontol.

[34] Robert AS, Cherepanov D, Palta M, Dunham NC, Feeny D, Fryback DG (2009). Socioeconomic status and age variations in health-related quality of life: results from the National Health Measurement Study. J Gerontol B Psychol Sci Soc Sci.

[35] Schmidt MI, Duncan BB, Azevedo e Silva G, Menezes A, Monteiro CA, Barreto SM (2011). Chronic non-communicable diseases in Brazil: burden and current challenges. Lancet.

[36] Simões EJ, Bouras A, Cortez-Escalante JJ, Malta DC, Porto DL, Mokdad AH (2015). A priority health index identifies the top six priority risk and related factors for non-communicable diseases in Brazilian cities. BMC Public Health.

[37] Stenholm S, Westerlund H, Head J, Hyde M, Kawächi I, Pentti J (2015). Comorbidity and functional trajectories from midlife to old age: the Health and Retirement Study. J Gerontol A Biol Sci Med Sci.

[38] Stevens A, Schmidt MI, Duncan BB (2012). Gender inequalities in non-communicable disease mortality in Brazil. Cienc Saude Coletiva.

[39] Taylor HL, Jacobs DR, Schucker B, Knudsen J, Leon AS, Debacker G (1978). A questionnaire for the assessment of leisure time physical activities. J Chron Dis.

